# New data regarding distribution of cattle ticks in the south-western Indian Ocean islands

**DOI:** 10.1186/1297-9716-44-79

**Published:** 2013-09-09

**Authors:** Frédéric Stachurski, Pablo Tortosa, Patrick Rahajarison, Stéphanie Jacquet, Amina Yssouf, Karine Huber

**Affiliations:** 1 CIRAD, UMR CMAEE, F-34398 Montpellier, France; 2 INRA, UMR 1309 CMAEE, F-34398 Montpellier, France; 3 FOFIFA-DRZV, BP 4, Antananarivo, Madagascar; 4 CRVOI, F-97490 Sainte Clotilde, La Réunion, France; 5 Université de La Réunion, 97490 Ste Clotilde, La Réunion, France

## Abstract

Recent studies have produced new insight into the origin and distribution of some cattle ticks in the south-western Indian Ocean islands. *Rhipicephalus appendiculatus*, introduced from Tanzania in 2002, is now well established on Grande Comore but has not yet reached the other islands of the archipelago (Mohéli, Anjouan and Mayotte). Only one of the two clades identified in Africa has settled so far. *Amblyomma variegatum*, which was not supposed to be able to persist in the Antananarivo region (1300 m) nor in other Malagasy regions of high altitude without regular introductions of ticks by infested cattle, is now endemic as a general rule up to 1600 m although other regions of lower altitude (1400 m) are still free of the tick. This species remains confined in a small area of the west coast on La Reunion Island. On the contrary, *Hyalomma dromedarii* could not settle on Madagascar where it was introduced in 2008 and *Rhipicephalus evertsi evertsi* is not yet present in Grande Comore despite regular introductions by infested cattle from Tanzania. A phylogeographic approach has been carried out at an intra-specific level for *A*. *variegatum*. This study has led to the identification of two main lineages, one covering all species distribution and one restricted to East Africa and the Indian Ocean area. These two lineages are in sympatry in Madagascar where a high genetic diversity has been described, whereas a lower genetic diversity is observed on other islands. These results seem to agree with the historical data concerning the introduction of the tick in the Indian Ocean area.

## Table of contents

1. Introduction

2. Successful ancient introductions

3. Failed recent tick introductions

4. Successful recent tick introductions

5. Alteration of tick distribution

6. Conclusions

7. Competing interests

8. Authors’ contributions

9. Acknowledgements

10. References

## 1. Introduction

Islands of the south-western Indian Ocean (SWIO) did not host ruminants (cattle, sheep and goats, deer) before human settlement regardless whether they split from Gondwana about 150 million years ago (Madagascar) or emerged de novo as volcanoes during the last 10 million years (Mauritius, Comoros, La Reunion). Cattle introduction occurred between the I^st^ and V^th^ centuries on Madagascar, in the VII^th^ century on Comoros, and in the XVII^th^ century on the Mascarene archipelago (Mauritius, Rodrigues and La Reunion). Archaeological research carried out on Madagascar led to the discovery of cattle bones dated from the V-VII^th^ centuries, the shape of the investigated skulls suggesting that *Bos taurus* was present at that period [1]. Zebus (*Bos indicus*) might have been bred on this island since at least the X^th^ century; it is, however, not clear whether these introductions were of Indonesian, Indian or African origin [2]. On Mauritius, cattle were introduced at least from the middle of the XVIII^th^ century for the needs of the sugarcane industry, the introduction of zebus from Madagascar being documented in the early XIX^th^[3]. Malagasy zebus were also introduced in La Reunion during the XVII^th^ and XVIII^th^ centuries and later crossbred with *Bos taurus* of European origin. Altogether, the available data suggest that the introduction of cattle on the islands of the region occurred first in Madagascar (*Bos taurus* followed by *Bos indicus*) and Comoros, and then in the Mascarene archipelago from Madagascar (*Bos indicus*) and from Europe (*Bos Taurus*).

Consequently, all cattle ticks present on these islands were introduced with their hosts after human settlement. As a result, some of the tick species have probably been present for centuries while others were introduced more recently. Tick species distribution may subsequently vary and evolve in response to cattle breeding characteristics, cattle movements or climate change. The latest data regarding the presence and distribution of cattle ticks in SWIO islands are presented below.

## 2. Successful past introductions

Despite the probable repeated introduction of ticks associated with centuries of regular cattle importation in Madagascar, cattle ticks were not described on the island before 1899. At that time, the presence of *A*. *variegatum* was reported both in Madagascar [4] and Mauritius [5] while it was not reported on La Reunion until 1949 [5]. *Boophilus microplus*, first described under other names [4,6] at the beginning of the XX^th^ century on Madagascar and other SWIO islands, is presently prevalent on all islands and in all environments. Regional cattle movements between East and Southern Africa and Madagascar lasted at least until the 1960s, causing a risk of introduction of new tick species (see below) and probably maintaining some level of gene flow between African and Malagasy tick populations. Cattle importation is still common in Grande Comore (see below) whereas no cattle have been introduced on La Reunion and Mauritius for decades, limiting the risk of tick species introduction. It is unclear whether these historical differences in cattle introduction resulted in the establishment of tick populations with different genetic and physiological characteristics. This has been recently studied for *A*. *variegatum* from different countries of SWIO in comparison with populations from Africa, where this species originates, and from the Caribbean islands, where it was introduced more than two centuries ago.

**Figure 1:**
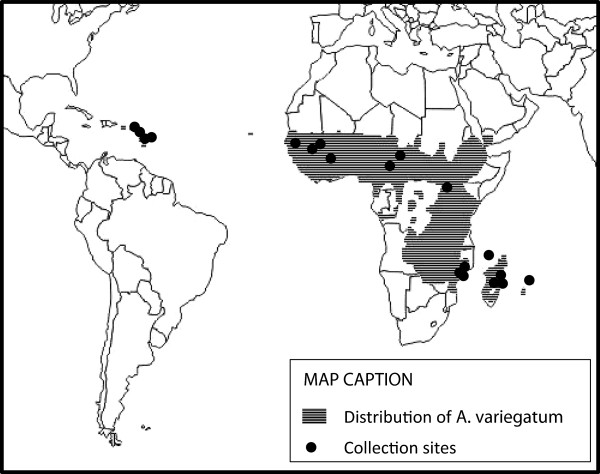
**Location of the *Amblyomma variegatum *samples used for the worldwide phylogeographic study.** The shaded area represents the present distribution of the tick and the black dots the sites where the ticks used in the study were collected.

**Figure 2:**
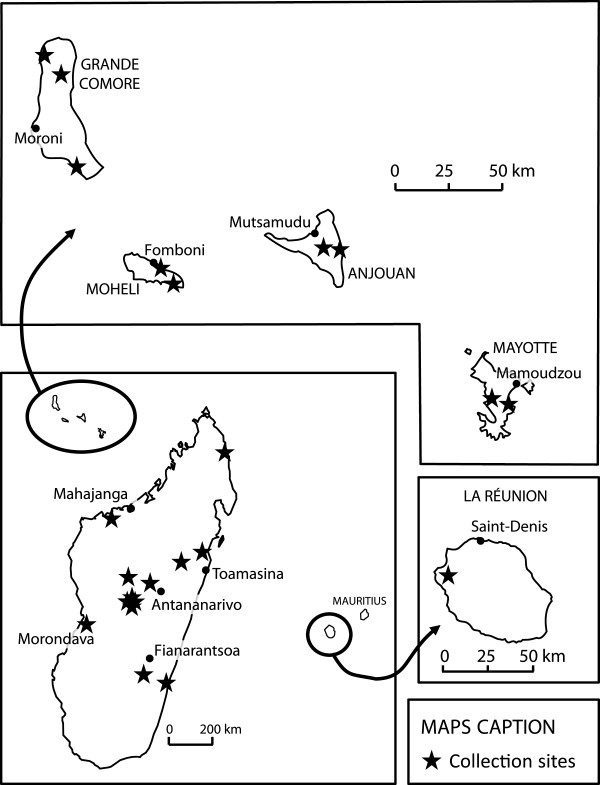
**Location of the *Amblyomma variegatum *samples used for the phylogeographic study restricted to the Indian Ocean area.** The black stars represent the sites where the ticks used in the study were collected.

One phylogeographic study based on two mitochondrial markers (12SrDNA and D-Loop) suggested that *A*. *variegatum* is divided into two distinct groups, an East African group and a West African group including Caribbean tick populations [7]. Our studies were conducted at two different scales, one at the worldwide level (Figure 1) and the other one including only tick samples from the Indian Ocean area (Figure 2). Ticks were collected exclusively from domestic cattle and identified according to Walker et al. [8]. Eight ticks per site were randomly selected for mitochondrial analyses. After DNA extraction, a 408 bp fragment of the mtDNA 16S ribosomal RNA and a 433 bp fragment of the mtDNA cytochrome b were amplified and sequenced. The worldwide study identified two major lineages, one covering the whole range of the species distribution while the other being restricted to East Africa and the Indian Ocean area (Huber, personal communication). Both large-scale studies hypothesize that *Amblyomma variegatum* originated from East Africa and diverged into two lineages along the Rift valley. The low level of genetic differentiation observed between East Africa and the Indian Ocean area suggests that *A*. *variegatum* was exported from East Africa to the SWIO islands. Such tick movements might actually still occur as there is currently cattle trade between East Africa and SWIO, anyway between Tanzania and Comoros islands.

At the local level, the two lineages are found in sympatry on Madagascar whereas the “worldwide” lineage is found quite exclusively on the other SWIO Islands (La Reunion, Mayotte and the Comoros Islands) (Jacquet, personal communication). A higher genetic variability (a high nucleotidic and haplotypic diversity and a positive Tajima D value) is observed on Madagascar, indicating large, stable population sizes. Whereas cattle ticks were not described on Madagascar before 1899, the introduction of *A*. *variegatum* is probably much older and concomitant with the cattle introduction on this island (see above). This ancient introduction followed by several successive re-introductions could have led to this high genetic diversity. A similar situation has been shown for the *Aedes aegypti* mosquito in Madagascar [9]. Conversely, a reduced genetic variability is observed on the other islands. This can be caused by a more recent introduction of the cattle tick to these islands and by repeated genetic bottlenecks (low number of ticks introduced, less re-introductions or repeated acaricide treatments), leading to smaller population sizes more susceptible to genetic drift. On Madagascar, it has been observed that the “worldwide” lineage was present throughout the island whereas the “East African” lineage was only detected in the center and the eastern part of the island which are the areas of Madagascar with the highest rainfall (Jacquet, personal communication). It has been hypothesized that the “worldwide” lineage could have a greater ecological plasticity allowing it to thrive in more variable conditions. This could explain the predominance of ticks belonging to this lineage on other islands of the Indian Ocean.

## 3. Failed recent tick introductions

Uilenberg mentioned repeated episodes of tick introduction on Madagascar without successful colonisation [10]. The ticks were generally discovered during cattle inspection at the quarantine in Toamasina harbour (east coast) on animals imported from South Africa: *Rhipicephalus appendiculatus* as well as other *Rhipicephalus* species, *Amblyomma hebraeum*, *Hyalomma marginatum rufipes* and *Hyalomma truncatum* were thus observed in these circumstances. Uilenberg also reported that goats were moved in 1963 from the harbour to a locality in the south of Madagascar where they were found infested by *H*. *truncatum* and *Rhipicephalus evertsi evertsi*. It seemed, however, that these species could not settle in spite of an apparently suitable climate.

Recently, five dromedaries were offered to Madagascar by the head of state of Libya. They arrived at Antananarivo airport in January 2008 with certificates guarantying an acaricide treatment before exportation and were immediately moved to a governmental residence in the south of the capital. One month later, the veterinarian in charge of the dromedaries brought ticks to the Veterinary Research Department (DRZV) of the National Centre of Applied Research for Rural Development (FOFIFA) where they were identified as *Hyalomma dromedarii* males. Two weeks later, the dromedaries were examined again by acarologists and found to be still infested by 40 male ticks. It is likely that, soon after their arrival in Madagascar, the female ticks had all engorged and dropped off in the pasture and premises where the animals were kept, in the vicinity of a dairy farm. However, four years later, no *H*. *dromedarii* had been reported either on the cows or on the dromedaries, which were in the meantime moved to the Antananarivo zoological gardens. It can thus be assumed that the local humid climate (more than 1200 mm of annual rainfall) and/or some other factors make the local environment unsuitable for this Sahelian tick.

In 2010, *R*. *e*. *evertsi* was found on cattle imported from Tanzania to Comoros, while it was absent on local cattle [11]. This suggests that this species either could not adapt to the local environmental conditions or that it was on its way to colonise Grande Comore. A proper tick survey on Comoros is necessary to check whether this species will succeed in colonising the archipelago.

## 4. Successful recent tick introductions

*Rhipicephalus appendiculatus*, a species of high veterinary importance in East Africa, was observed for the first time in Mauritius in 1980 although probably introduced from South Africa some 20 years earlier [5]. More recently, this species was introduced on Grande Comore through cattle importation from Tanzania in 2002, leading to numerous mortalities due to East Coast Fever (ECF) during the following months [12]. Interestingly, the origin of cattle importation to Comoros has switched from Madagascar to Tanzania following a free trade bill signed in 2000. This switch was associated with the emergence of several epidemics (ECF, Chikungunya, Rift Valley Fever - RVF - and Dengue), some of which spread to other islands of the region (Chikungunya and RVF). This is a documented episode emphasizing the importance of trade-associated cattle movements in the emergence of zoonosis [11-15].

**Figure 3:**
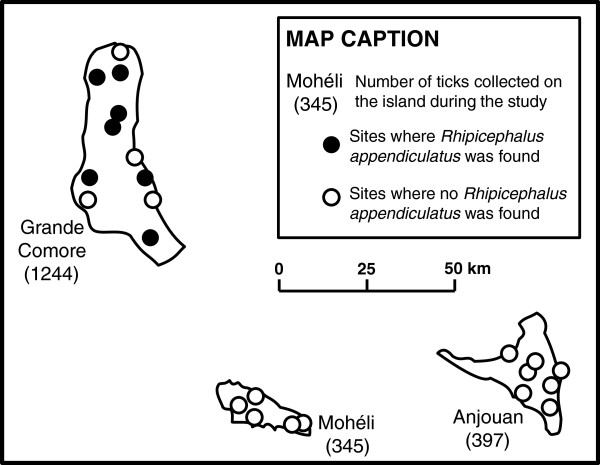
**Distribution of *Rhipicephalus appendiculatus *in the Union of the Comoros according to the survey carried out in January-February 2010 (adapted from Yssouf et al. 2011 [**[11]**]).** The number of ticks of all genera collected appears in brackets near the name of each island. The black dots represent the sites where *R*. *appendiculatus* was found, the white dots the sites where only *R*. *microplus* and/or *A*. *variegatum* were found.

A survey was carried out in January and February 2010 on 34 sites distributed over 16 of the 17 districts of Comoros. Five animals were randomly selected on each site and visually examined. All ticks were sampled in low infested animals, and up to 150 ticks on heavily infested animals. Ticks were found on 23 of the inspected sites, distributed on the 3 islands. The collected ticks were identified morphologically with the help of the key provided by Walker et al. [8]. This study showed that *R. appendiculatus* had established throughout Grande Comore. It was, however, absent from the two other Comorian Islands: Anjouan and Mohéli (Figure 3). The species was also absent from the French administrated neighbouring Island of Mayotte. This limited distribution was in accordance with the absence of ECF in Mohéli, Anjouan and Mayotte [11]. The inter-island livestock movements can explain this tick distribution since most cattle move from Tanzania, Moheli or Anjouan towards Grande Comore, where important cattle slaughtering occurs during traditional weddings called “grands mariages”. Illegal livestock transport to Mayotte (located 70 km south of Anjouan) occurs essentially from Anjouan and not from the other islands of the Union of the Comoros and concerns mainly goats. A phylogenetical analysis of *R*. *appendiculatus* showed that two clearly distinct clades were sampled on cattle imported from Tanzania while only one of them was found on locally reared cattle, suggesting that one group is perhaps more adapted to the locally encountered environment. It is noteworthy that, the successful *R*. *appendiculatus* clade was reported to be more competent for *Theileria parva* transmission, causing ECF, than the other unsuccessful clade [11]. Data summarised above confirm that the islands of the region are subjected to repeated tick introductions, but for various reasons only some of the tick species or sub-species succeed in colonising new areas and establishing self-sustained populations. This suggests that dispersion capacities of ticks are actually high, but that local environmental conditions play a major role in limiting tick distribution [6].

As stated above, *R*. *appendiculatus* is present on Mauritius but not *Theileria parva*, the pathogen responsible for ECF. In 1980, the tick was observed only on two farms located on the south of the island. In 2009, a *R*. *appendiculatus* male was collected in a cattle farm in the north of the country (Grande Daube), highlighting the need for a proper survey of tick distribution on the island. Since the vector seems to have been established on that island, introduction of animals coming from Comoros should be as restricted and controlled as that from the African mainland. Madagascar used to export cattle to Comoros until 2000 but the reverse seemingly never occurs, at least officially. Whatever the case, this recent settlement and the consecutive mortalities, which are going on 10 years later, should prompt the veterinary authorities of all the islands of the region to strictly control animal movements and use adequate quarantine containment facilities when cattle introductions are required or decided [6].

## 5. Alteration of tick distribution

On La Reunion, *A*. *variegatum* seems to becoming scarce. Barré and Morel found it from Saint-Denis (north) to l’Etang Salé (south-west) up to 300 m altitude [5]. In 2001, a survey confirmed its presence on the island but with a distribution restricted to the western portion of the previous distribution area. However, some specimens were collected at 900 m altitude. In 2009–2010, cattle and goats on 22 farms of the west coast were examined and *A*. *variegatum* were observed at only 2 sites, La Saline and Saint-Leu, where only 59 ticks were collected on the 70 examined cattle, all being “Moka”, i.e. Malagasy zebus or crossbred with that breed (Grimaud, personal communication). On Mauritius, similar changes did not occur. The tick is still present throughout the island, although mainly on lowland pastures, parasitizing cattle and deer as previously observed [5].

**Figure 4:**
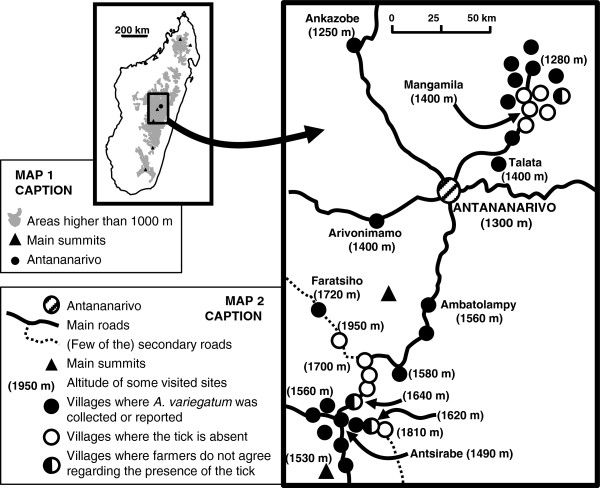
**Distribution of *Amblyomma variegatum *on the central high plateau of Madagascar according to tick collection and surveys carried out in 2008 and 2010.** The grey areas in the small map represent the areas higher than 1000 m, and the dot the location of the capital city, Antananarivo. In both maps, the triangles represent the main summits. On the close-up map of the central high plateau, the plain and dash lines represent the main and secondary roads, respectively; the numbers in brackets, the altitude of some of the visited sites; the black dots, the villages where *A*. *variegatum* was collected or reported by the polled farmers; the white dots, the villages where the tick is absent; and the half black-half white dots, the villages where the polled farmers did not agree regarding the presence of the tick.

In Madagascar, Uilenberg et al. mentioned that « in Antananarivo area (1100 to 1400 m), *A*. *variegatum* is not rare during the rainy season, along the trails followed by the herds coming from the western regions and walking towards the abattoirs of the capital. The tick is absent or rare where these herds are not passing through, and it is uncertain that it can persist in Antananarivo region without continuous introduction from the western provinces » [4]. In 2008, tick collections were done in various villages of the high plateau. It was determined that the tick was unknown in the Mangamila area (1400 m) but more or less abundant in Ankazobe (1250 m), Arivonimamo (1400 m) and Talata (1400 m) (Figure 4). In 2010, a survey was carried out on 80 villages of the Mangamila area, 70 km north from Antananarivo, and in the Antsirabe area. Discussions were organised in each village with farmers to whom the two cattle ticks of Madagascar (*B*. *microplus* and *A*. *variegatum*), kept in alcohol, were shown. People were asked whether they knew these ticks and whether they were infesting cattle of the village. According to the answer, more details were asked for, such as the date of the first observed infestation, the seasonality of the infestation, the presence of the ticks in the neighbouring villages or areas… *B*. *microplus* was known by all farmers. As far as *A*. *variegatum* is concerned, three answers were obtained: “the tick is present here”; “the tick is not here but we know it and have seen it in other villages/cattle markets”; “we never saw this tick”. Only on a few occasions, in some places at the limit between tick-free and tick-infested areas, farmers did not agree regarding the presence of *A*. *variegatum* (Figure 4). It appeared that the Mangamila area (about 200 km^2^) remains free of *A*. *variegatum* although the tick has infested some villages at the border of the free area for sometimes 20–40 years. It is not clear why the tick has not yet settled in the free area despite regular movement of infested animals coming from endemic villages. Climate and vegetation of both (infested and free) areas are similar and a field experiment revealed that at least three free stages of the tick (unfed and engorged nymphs, unfed adults) could overwinter (Stachurski and Rahajarison, personal communication). The low ruminant density, the absence of pasture sharing between tick-free and tick-infested villages, the stated acaricide treatment of all introduced animals could maintain such a tick-free situation, although an infestation is clearly to be feared in the future.

The distribution area has extended to the Ambatolampy and Antsirabe areas (central high plateau) where *A. variegatum* is now present in villages below 1600 m, and perhaps up to 1700 m in some isolated places like Faratsiho (Figure 4). There are, however, still some tick-free pockets, in the highest and therefore coldest areas. As the Antsirabe area is the dairy production zone of Madagascar, where exotic cattle (pure or crossbred of Friesian or red and white Norwegian) are reared, important losses due to cowdriosis are a clear threat. Some field veterinarians have already reported cowdriosis although this was never confirmed in the laboratory.

The presence of *A*. *variegatum* up to 1600 m on Madagascar and on the Adamawa highland of Cameroon [16] is an indication that the absence of the tick in high pastures of La Reunion is probably not due to the inability of the species to survive in such conditions. Very regular tick control, good monitoring of cattle movements between farms, lack of trade between the “Moka” herds and the other livestock (Friesian, Limousin) may be sufficient to limit the distribution of this tick on that island.

## 6. Conclusions

Data analysis demonstrates that the degree of success of tick colonisation of new areas depends on a range of factors (e.g. environmental, climatic, anthropic and genetic). Recent observations regarding cattle tick distribution in SWIO show that, despite the fact that some tick species have been established on islands for centuries, alterations in their distribution continue to occur mainly associated with host movements and/or climate change. A new vector species (*R*. *appendiculatus*) was recently introduced to Grande Comore and may potentially settle on the neighbouring islands, where it could lead to significant losses. As far as *A*. *variegatum* is concerned, the recent extension of its distribution in Madagascar could also lead to major losses due to cowdriose or dermatophilosis in areas where dairy cattle of European breeds are reared. Several apparent failures in establishment of new tick populations were also reported: only one of the two *A*. *variegatum* lineages has a wide distribution in the region; only one of the two *R*. *appendiculatus* clades settled at present on Grande Comore; *R*. *e*. *evertsi* has not succeeded to settle on Madagascar or Comoros despite several introductions. Yet, this apparent inability of certain tick species or subspecies to colonise additional islands or particular environments should not cause postponement in the establishment and strict enforcement of safety measures against introduction of new tick species and their associated tick-borne pathogens to the SWIO islands.

## 7. Competing interests

The authors declare that they have no competing interests.

## 8. Authors’ contributions

FS, PT and KH wrote the manuscript. FS and PR collected the data and the ticks in Madagascar. PT and AY carried out the work on *R*. *appendiculatus* in Comoros. KH and SJ did the analysis regarding *A*. *variegatum* phylogeographic. All authors read and approved the final version.
